# Severe acute respiratory syndrome coronavirus 2 as an atypical cause of Bell's palsy in a patient experiencing homelessness

**DOI:** 10.1017/cem.2020.418

**Published:** 2020-06-08

**Authors:** Shaun Mehta, David Mackinnon, Sahil Gupta

**Affiliations:** * Division of Emergency Medicine, Department of Medicine, University of Toronto, Toronto, ON; † Department of Emergency Medicine, St. Michael's Hospital, Toronto, ON; ‡ Department of Family and Community Medicine, University of Toronto, Toronto, ON

**Keywords:** Administration, COVID-19, infectious diseases, microbiology, public health

## INTRODUCTION

Bell's palsy is an idiopathic paralysis of the facial nerve (cranial nerve VII) and is a fairly common presentation in the emergency department (ED) setting. Although there are a variety of etiologies, it has been postulated by some to be secondary to a viral infection, commonly attributed to herpes simplex virus. We present a case of Bell's palsy that we attribute to severe acute respiratory syndrome coronavirus 2 (SARS-CoV-2).

## CASE PRESENTATION

A 36-year-old man presented to the ED with concerns of ongoing numbness, tingling, and weakness to his right face. His past medical history was unremarkable, and he was on no medication.

Before developing neurologic symptoms, he had experienced subjective fever and chills for three days. He also had generalized myalgias with moderately intense pain to his right neck, radiating to the angle of the jaw.

His triage vitals showed a blood pressure of 125/75, heart rate of 61, respiratory rate of 18, oxygen saturation of 99%, and temperature of 36.3°C. On exam, he had an obvious right-sided facial droop with incomplete closure of his right eye and no grossly perceptible movement of his right forehead. He also had decreased sensation along the right side of his face from the upper forehead to his jaw. The remainder of his cranial nerve testing was normal. Sensation, strength, reflexes, and tone were unremarkable in the trunk and extremities. His gait was normal. The patient also had tenderness on the right neck along his cervical chain and just below the angle of the mandible. His neck was supple. Routine laboratory findings were non-contributory with white blood cells 3.83, neutrophils 1.5, and lymphocytes 1.4.

As the patient was experiencing focal neck pain, cerebral vascular imaging was done to rule out an alternative pathology. A computed tomography (CT) angiogram of the head and neck showed no acute abnormalities.

A diagnosis of Bell's palsy was made, and he was prescribed a course of oral prednisone as well as eye lubrication. At our hospital, the patient met coronavirus disease 2019 (COVID-19) testing criteria because he had recent viral symptoms and was experiencing homelessness.

Because he was unable to self-isolate at his current shelter, he was referred to an isolation shelter. His COVID-19 nasopharyngeal swab came back positive and he was subsequently transferred to a COVID-19 isolation shelter. The test was performed using the Altona RealStar® SARS-CoV-2, RT-PCR Kit 1.0.

## DISCUSSION

The international COVID-19 pandemic has been at the forefront of healthcare for several weeks.

Screening criteria for COVID-19 for most EDs, albeit heterogeneous and dynamic, include influenza-like symptoms with some recognition of more atypical symptoms, such as diarrhea. Despite rigorous efforts to catch potential COVID-19 patients at triage, a large number of infections remain either asymptomatic or underrecognized.^1^

**Figure 1.**
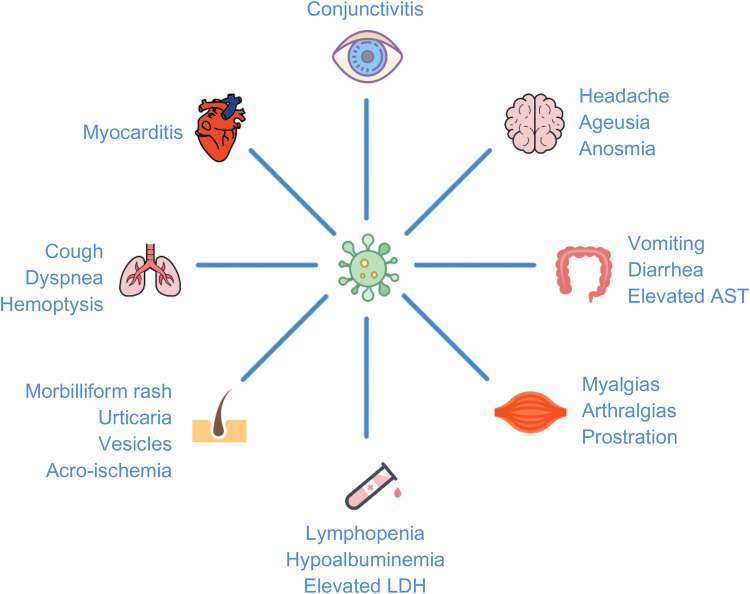
Systems-based approach to typical and atypical COVID-19 signs/symptoms.^2–5^

There have been many atypical presentations of COVID-19 described.^2–5^ They range from mild presentations, such as anosmia, nausea, vomiting, diarrhea, and unexplained tachycardia, to life-threatening conditions, such as respiratory failure, sepsis, and myocarditis (Figure 1). Based on a literature review and recent online case reports, this is the first reported case of Bell's palsy potentially attributable to SARS-CoV-2. Indeed, it is possible that our patient had asymptomatic COVID-19 as well as Bell's palsy from an alternative etiology, yet this scenario is unlikely given the patient's viral prodrome.

COVID-19 screening and testing criteria differ widely across Canada. For example, as of April 2020, the screening criteria in Alberta offered testing to anyone with fever, cough, shortness of breath, runny nose, or sore throat. Meanwhile, in Ontario, testing has been limited primarily to: 1) patients being admitted to the hospital, 2) those living or working in congregate settings (e.g., nursing homes and homeless shelters), and 3) healthcare and other essential workers. Testing can be done at the discretion of the treating physician; however, clear and specific standards are helpful to establish practice consistency.

Our case report has implications for personal protective equipment (PPE) and cohorting patients within the ED. Although most screening strategies are conservative, some COVID-19 patients will not be captured through standard triage protocols. As a result, these patients may not be isolated in the ED or examined with appropriate PPE. Our case suggests that certain presentations, which have a potential viral etiology (including Bell's palsy) and would otherwise screen negative, should be considered as suspected COVID-19.

If this patient had not been living in a shelter, he would not have met our testing criteria. Without our vigilant testing of patients experiencing homelessness, he would have been treated and discharged back to his shelter, potentially exposing many others to SARS-CoV-2. This case gives credence to the continued need to test widely and broadly, especially for high-risk groups such as patients experiencing homelessness, in order to appropriately cohort patients within the ED and in the community.

## CONCLUSION

We present a case of Bell's palsy likely attributable to SARS-CoV-2. As we continue to learn about COVID-19, we will certainly discover more atypical signs and symptoms. Our case highlights the need for maintaining a high index of suspicion in a broader range of presentations. This will help inform isolation strategies, both while in the ED and upon discharge. This approach is underscored in high-risk groups, such as patients experiencing homelessness where even one missed case can have significant epidemiological consequences.
